# Investigation of the effects of 3D printing parameters on mechanical tests of PLA parts produced by MEX 3D printing using Taguchi method

**DOI:** 10.1038/s41598-025-98832-0

**Published:** 2025-04-29

**Authors:** Özgür Verim, Omar Saeed, Mohamed Hamdy Eid, Samy F. Mahmoud, Dalia I. Saleh, Abdallah Elshawadfy Elwakeel

**Affiliations:** 1 Department of Mechanical Engineering, Faculty of Technology, Afyon Kocatepe University, 03200 Afyonkarahisar, Turkey; 2 Doctoral School of Environmental Science, Hungarian University of Agriculture and Life Sciences (MATE), Páter Károly u. 1, Gödöllő, 2100 Hungary; 3 Institute of Environmental Management, Faculty of Earth Science, University of Miskolc, Miskolc- Egyetemváros, 3515 Hungary; 4 Geology Department, Faculty of Science, Beni-Suef University, Beni-Suef, 65211 Egypt; 5 Department of Biotechnology, College of Science, Taif University, Taif City, Saudi Arabia; 6 Department of Chemistry, College of Science, Taif University, Taif City, Saudi Arabia; 7 Department of Agricultural Engineering, Faculty of Agriculture and Natural Resources, Aswan University, Aswan, 81528 Egypt

**Keywords:** Infill density, Layer thickness, Raster angle, Printing speed, Wall thickness, Taguchi analysis, Mechanical test, PLA, MEX, Mechanical engineering, Mechanical properties

## Abstract

With the widespread use of the Material Extrusion method in the 3D printing industry, it has become important to determine the optimum printing parameters to improve the mechanical characteristics of Polylactic Acid material according to the place of use. It is important to choose the right press parameters according to the forces to which the material is subjected. In research, we examined the impacts of 3D printing parameters such as Layer Thickness, Infill Density, Raster Angle, Printing Speed, and Wall Thickness on mechanical tests covering tensile, compression, flexural, impact, hardness, and surface roughness. We employed Taguchi analysis to streamline the number of experiments and determine the optimal printing parameters to maximize mechanical performance. Five samples were determined for each experimental design measurement. According to the results of the research, Layer Thickness and Infill Density parameters were found to be very important parameters in mechanical tests according to the effect and contribution ratios. The effect ranking of the Infill Density parameter on tensile, compressive and impact strength was the best (1) and the contribution rate was the highest (55.21%, 80.86% and 63.61%). In flexural strength, the order of effect and contribution rate (25.66%) were found in the 2nd place. The effect of Layer Thickness parameter on impact, hardness and surface roughness tests was found to be significant (2,1 and 1). The effect values in tensile and compression tests were also found to be moderate (3). Considering the efficiency levels and contribution rates for the other parameters, the order of importance can be listed as Wall Thickness, Raster Angle and Printing Speed. With the help of this study, the importance levels of the parameters during the formation of parts under combined forces were determined and presented to the literature.

**Supplementary Information:**

The online version contains supplementary material available at 10.1038/s41598-025-98832-0.

## Introduction

In the industrial sector, additive manufacturing facilitates the utilization of three-dimensional (3D) intricate and conceptual objects, which are typically produced through more complex and time-consuming methods in traditional manufacturing processes, within shorter time frames and at reduced costs. This technology enhances both the speed and adaptability of the design and production processes^1^. Additive manufacturing methods are increasing their weight in the manufacturing sector day by day compared to traditional manufacturing. For instance, additive manufacturing is used in many fields such as aerospace, automotive, medicine, textile, education, electrical-electronics, architecture, construction, food, etc.^2,3^ This is due to the widespread availability of complex parts that may be easily made without human intervention, the realization of custom-made production, and the availability of affordable prices^4,5^ outline the benefits of additive manufacturing compared to traditional manufacturing methods as follows: production of complex parts without joints, lower material and labor costs, less energy demand, good surface quality, easy control of process temperature, low process complexity, fast production process, production close to actual dimensions, short delivery time, lower total cost compared to conventional production, etc.

Additive manufacturing can be broadly categorized based on production methods and strategies into several types: MEX, stereolithography (SLA), selective laser melting (SLM), direct ink writing (DIW), selective laser sintering (SLS), laminated object manufacturing (LOM), electron beam melting (EBM), and binder jetting (BJ)^2,6^. Among these, MEX is one of the most widely used methods due to its user-friendliness, accessibility, rapid production speed, affordability of equipment and materials, and capability to process thermoplastic polymers. MEX technology is a material extrusion process reliant on thermoplastic materials including Polylactic acid (PLA), Nylon, Acrylonitrile butadiene styrene (ABS), Polystyrene (PS), Polycarbonate (PC), Polyether-ether-ketone (PEEK), extensively utilized in the industry. This technology has evolved to a point where low-cost 3D printers are now prevalent in the industry^7,8^. Compared to other thermoplastics, PLA is favored in various sectors such as electronics, automotive, and mechanics owing to its low ductility, high tensile strength, and biodegradable nature^8,9^.

**Fig. 1:**
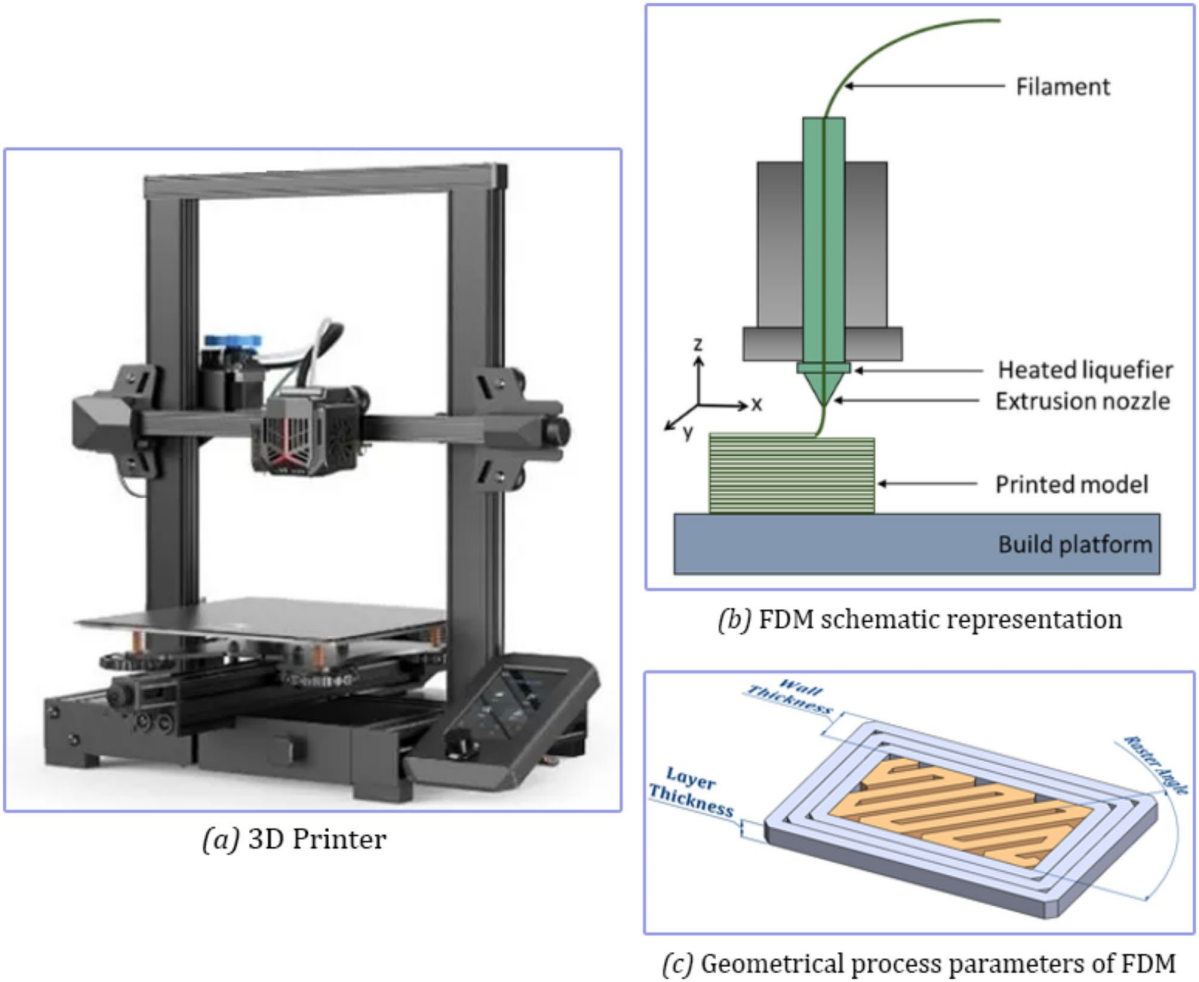
(**a**) 3D printer used in the study, (**b**) MEX method, (**c**) some of the parameters used in the MEX method.

Additive manufacturing technology is also commonly known in the literature as 3D printing (3DP), Rapid Prototyping (RP) and Solid Freeform Fabrication (SFF)^10,11^. This technology, which emerged after the 1980s, enables the production of parts from scratch without the need for any mold system. For 3D printing, a 3D solid model of the part or assembly must first be created with the assistance of computer-aided design (CAD) programs. In order for the 3D model to be recognized by the 3DP device, it must first be converted to Stereolithography (STL) file format and G codes must be created with slicing programs^12^. In a typical 3D printer, an object is formed by depositing melted material layer by layer, with an extruder that moves along three axes (x, y, and z) and provides single or multiple feed options (Fig. 1b). As the MEX method has become increasingly popular in the 3DP industry, optimizing printing parameters to enhance the mechanical properties of the material has gained importance. The key process parameters that significantly affect the mechanical quality of 3D-printed objects include layer thickness, infill density, printing speed, raster angle, and wall thickness^13–16^. Apart from these, there are many parameters that are considered as details and affect the mechanical structure^13,17^. The main purpose of all the parameters determined is to produce a mechanically more stable structure in the shortest time by using less material. In the literature, each author has tried to achieve their targeted results by using certain 3DP process parameters according to the scenarios they have determined^4,5,18–24^. A number of tests are conducted to explore the mechanical behavior of the parts created by the MEX method. Thanks to these tests, the interactions between process parameters can be easily determined. While there are no published standards for the production of thermoplastic parts by the MEX method, there are numerous standards for mechanical testing of plastics in general^17^. The general mechanical tests used in the MEX method and valid for plastics can be listed as tensile, compression, flexural, Charpy impact, hardness and surface roughness tests. Extensive research has been carried out to examine the impacts of 3DP parameters on the flexural, tensile, compression, impact, hardness, and surface roughness properties of thermoplastic PLA materials. The large body of literature on this subject provides a thorough understanding of how various process parameters influence these properties. Nevertheless, the comprehensive assessment of the collective influences of 3DP parameters on the mechanical characteristics of thermoplastic PLA components has not been widely explored. In addition, as it is known, mechanical parts are generally subjected to integrated loads and their strength under combined loads (for example, compression, tension and bending together) comes to the fore. This study will guide us in this regard. Our research aims to fill such a work gap and to provide a basis for those who want to work in this field. The objective of this research is to investigate the effects of key process parameters on various mechanical tests, including tensile, compression, flexural, Charpy impact, hardness, and surface roughness tests, conducted on parts manufactured via the MEX method. Taguchi L16 orthogonal array was used to plan the experimental design and experiments, and statistical studies were carried out accordingly. This study focused on five 3DP process parameters: layer thickness, infill density, printing speed, raster angle, and wall thickness. To analyze the interactions between these parameters, Signal-to-Noise (S/N) ratio, analysis of variance (ANOVA), and regression analysis were utilized.

With this study, the behavior of 3d printing parameters under different mechanical tests for the design of 3d printing parts under different loads was examined and a preliminary information was tried to be presented to the designer. The designer will be able to realize the design according to the importance levels and effectiveness of the printing parameters according to the working condition of the part in the system.

## Material and methods

### Experimental design and optimization

The research employed the Taguchi method for both experimental design and analysis, which consists of six steps: (1) defining the parameters and their respective levels to be tested, (2) choosing a suitable orthogonal array, (3) assigning the parameters and their levels to the array columns, (4) executing experiments based on the orthogonal array, (5) computing signal-to-noise ratios, and (6) conducting validation experiments. The 3DP process factors and their levels used in the Taguchi analysis are illustrated in Fig. 1c and detailed in Table 1. Each parameter was evaluated at four different levels.

**Table 1:** 3DP process parameters and their levels.

Parameter	Description	Level
Layer thickness (mm)	In the MEX method, the print is formed by stacking planar layers on top of each other. The thickness of a single plane layer is represented here	0.1, 0.15, 0.2 and 0.25 were used in the study
Infill density (%)	Percentage ratio of part solid to whole in the final print	Values of up to 5% can be applied for lightweight and non-strength parts, while up to 100% is suitable for parts requiring greater strength. In this study, the levels used were 25%, 50%, 75%, and 100%
Printing speed (mm/s)	Speed of the nozzle connected to the extruder during printing	40, 50, 60 and 70 were determined as the values frequently used in studies
Raster angle (°)	It can be described as the angle that the nozzle makes with the x-axis during the production of the inner part of the part in printing. See also Fig. 1c	0, 30, 45 and 75 degrees were determined as frequently used values in studies
Wall thickness (mm)	It is the thickness of the solid part that forms on the outermost part of the part during printing. See also Fig. 1c	In the study, wall thickness was determined as 0.6, 0.9, 1.2 and 1.5 mm

Design of Experiments (DOE) is a systematic approach used to assess how input process parameters influence one or more output responses. By systematically varying input factors, DOE helps determine optimal process parameters and identify key factors and their best levels to meet performance targets. Various DOE methods, including the Taguchi method^19^, full factorial designs, ANOVA^25^ and fuzzy logic^26^, are commonly employed to optimize MEX process parameters. The Taguchi method differs from traditional techniques by focusing on quality design during the product and process design phases, whereas traditional methods typically concentrate on quality control and inspection during or after manufacturing. The Taguchi method uses ANOVA or the S/N ratio to achieve desired results. ANOVA evaluates variance among different groups to understand the overall impact of individual production parameters on final characteristics, while the S/N ratio method examines variation in responses relative to nominal values under various noise conditions. The results are then analyzed by adjusting process parameters based on average output responses. ANOVA is typically used to analyze the percentage influence of each parameter on individual outputs, whereas the S/N ratio method is applied when optimizing a process with multiple responses. This study aims to explore the optimal conditions for Infill Density, Layer Thickness, Printing Speed, Raster Angle, and Wall Thickness by using the S/N method, given the goal of optimizing a multi-response process. In this approach, a loss function quantifies the difference between experimental values and target values, derived from the average mechanical properties obtained. This discrepancy is then converted into the S/N ratio, which allows for the evaluation of the balance between quality and variability^24^. Depending on the context, there are three types of S/N ratios: (a) lower is better, used for minimizing performance characteristics; (b) nominal is better; and (c) larger is better. In this study, a “Larger is better” S/N ratio as defined in Eq. (1) was used to maximize tensile, compressive, flexural, Charpy impact strengths and hardness values. In addition, for surface roughness, the “Smaller is better” S/N ratio was used in Eq. (2).

1 $$S/N = - 10\log \left[ {\frac{1}{n}\mathop \sum \limits_{i = 1}^{n} \frac{1}{{Y_{i}^{2} }}} \right]$$

2 $$S/N = - 10\log \left[ {\frac{1}{n}\mathop \sum \limits_{i = 1}^{n} Y_{i}^{2} } \right]$$

In this context, *n* represents the number of data points, and *Y* denotes the value of the *i*th data point. The parameters that yield the highest S/N ratio are considered the optimal ones, as they offer the best strength resistance characteristics. Using a comprehensive factorial design with four levels for Layer Thickness, Infill Density, Printing Speed, Raster Angle and Wall Thickness, samples will need to be produced and tested to cover all possible permutations. However, such an approach is considered inefficient in terms of both time and filament consumption. Using the Taguchi method, an L16 orthogonal array was selected as an alternative. In essence, this means that instead of doing 1024 experiments, only 16 experiments need to be done. Details of Taguchi’s sixteen experiments are summarized in Table 2, together with the relevant process parameters.

**Table 2:** Experimental design using the L_16_ orthogonal array.

Test Number	Layer thickness (mm)	Infill density (%)	Printing speed (mm/s)	Raster angle (°)	Wall thickness (mm)
1	0.10	25	40	0	0.6
2	0.15	25	50	30	0.9
3	0.20	25	60	45	1.2
4	0.25	25	70	75	1.5
5	0.25	50	40	30	1.2
6	0.20	50	50	0	1.5
7	0.15	50	60	75	0.6
8	0.10	50	70	45	0.9
9	0.15	75	40	45	1.5
10	0.10	75	50	75	1.2
11	0.25	75	60	0	0.9
12	0.20	75	70	30	0.6
13	0.20	100	40	75	0.9
14	0.25	100	50	45	0.6
15	0.10	100	60	30	1.5
16	0.15	100	70	0	1.2

### Specimen preparation and mechanical testing

3D models of the mechanical test pieces were created with the computer-aided design program SolidWorks 2023 (Dassault Systèmes). The 3D model, which was converted into STL format with the CAD program, was simulated for printing with the Simplify 3D program and converted into G code. The designed test samples were produced with Creality Ender3 S1 Pro 3D printer in accordance with PLA printing parameters (Fig. 1a)^20^.

PLA was chosen as the printing material for this research owing to its advantages over ABS^27^. PLA is a biodegradable thermoplastic polyester derived from renewable resources. It offers lower toxicity and can be processed at lower temperatures compared to many other thermoplastics^28^.

### Mechanical testing methods

**Fig. 2:**
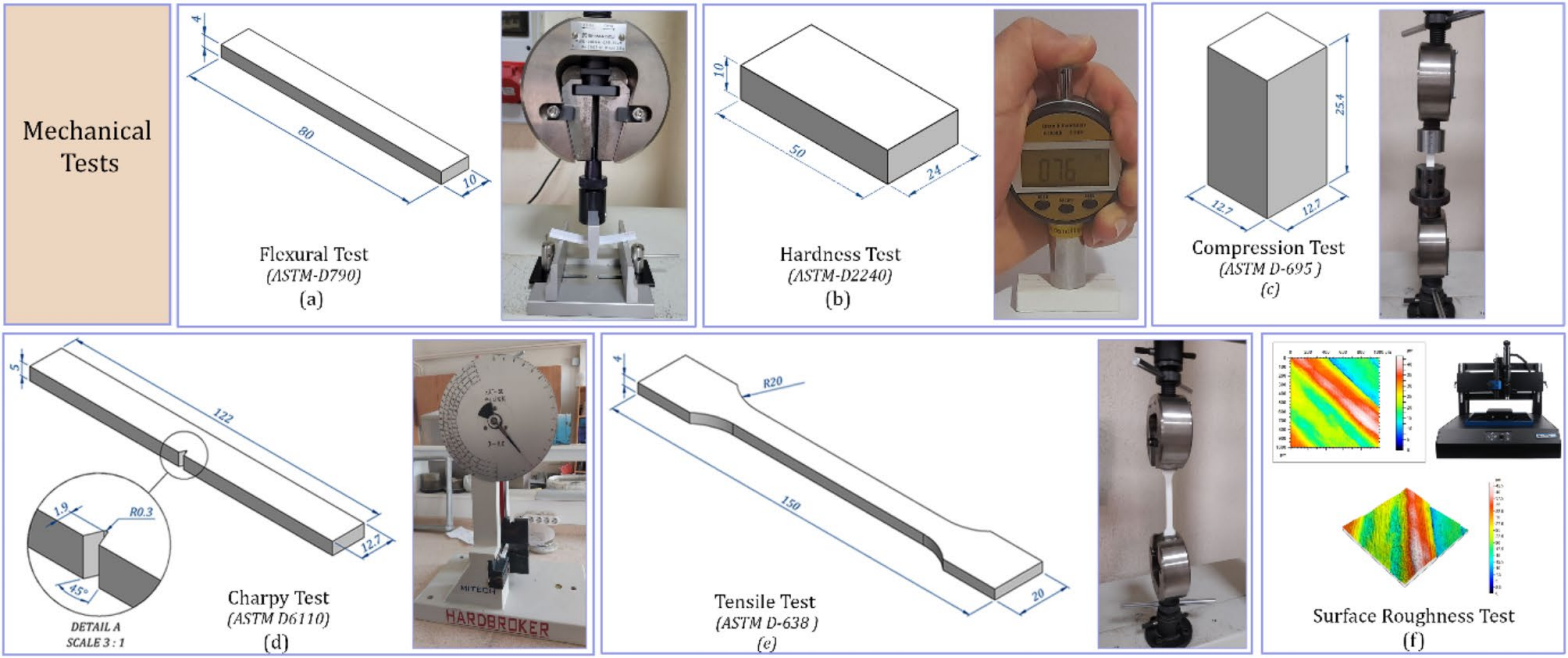
Test methods used in the research.

While there are no standards for the production of thermoplastic parts by the MEX method and their mechanical testing, there are many standards for the mechanical testing of plastic parts. This research will explore in detail the influences of fundamental process parameters on the mechanical traits of parts made using the MEX method. The specimen geometry dimensions of the mechanical test methods and their contents to be used in the research are shown in Fig. 2. The MEX method can directly print the specimen geometry required for any mechanical test method. Since the MEX method produces final dimensions, no post-processing is required.

The dimensioning and testing procedures of the specimens produced with PLA material were conducted in accordance with the relevant standards; ASTM D-638 standard for tensile test^18^, ASTM D-695 standard for compression test^18^, ASTM-D790 standard for flexural test^29^, ASTM D6110 standard for Charpy impact test^19^, ASTM-D2240 for hardness measurement^30^. In mechanical testing of polymer materials, parameters such as yield strength, modulus of elasticity, and tensile strength are used to characterize the material. Since this research focuses on material strength, tensile strength will be the primary parameter of interest.

### Tensile test setup

Test specimen dimensions are shown in Fig. 2e. Five samples were prepared for each design point according to the standards. Shimadzu brand tester was used in the tensile test and the displacement rate of the device was taken as 0.1 mm/min. Each specimen underwent a quasi-static tensile test conducted at room temperature. The specimen connection to the testing machine is shown in Fig. 2e. The ultimate tensile strength was considered in the tensile test procedure. The engineering maximum tensile strength equation for homogeneous materials is given in Eq. 3.

### Compression test setup

In the compression test, the compression strength is defined as the maximum load that the object can endure under the given loading conditions before failure. The test was evaluated on five specimens and the compression test was performed on a Shimadzu brand tester with a displacement rate of 0.1 mm/min. The compression test piece and assembly connected to the test machine are presented in Fig. 2e. The maximum engineering compressive strength for homogeneous materials is given in Eq. (3).

3 $$Tensile \, and \, Compression \, Strength \, \left({\sigma }_{t,c}\right) =P/A$$

The parameters in the equation; ${\sigma }_{t}$ represents the tensile strength (MPa), ${\sigma }_{c}$ represents the compressive strength (MPa), P represents the force (N) and A represents the cross-sectional area of the part before starting the test.

### Flexural test setup

PLA material’s flexural strength was evaluated using a Shimadzu test machine, with a displacement rate of 0.1 mm/min applied by the upper jaw during the test. Five specimens were tested, and the setup for the flexural test of thermoplastic PLA specimens is illustrated in Fig. 2a. For homogeneous elastic materials, the maximum flexural stress is observed at both the outer surface and the center of the specimen. The equation for calculating flexural strength is provided below (Eq. 4).

4 $$Flexural \, Strength \left({\sigma }_{f}\right)=3PL/2b{d}^{2}$$

The parameters in the equation are defined as follows: σ represents the stress in the outer fibers at the midpoint (MPa), *P* is the load at a specific point on the load–deflection curve (N), *L* denotes the support span (mm), *b* is the width of the tested beam (mm), and *d* is the depth of the tested beam (mm).

### Charpy impact test setup

Charpy impact tests were accomplished on five specimens per group using a MITECH Hardbroker XJJ-50 impact tester equipped with a 4-Joule hammer. The tests were conducted under controlled laboratory conditions, with a relative humidity of 50% and a temperature of 23 °C. The experimental setup for the Charpy impact test specimen is shown in Fig. 2d. Each specimen was impacted by a controlled hammer pendulum traveling at a velocity of 3.75 m/s. The impact energy was calculated by measuring the difference in potential energy between the hammer’s initial and final positions. This energy difference was then used to determine the Charpy impact strength, as given by Eq. (5):

5 $$Equations= \left\{\begin{array}{l}Fracture \, energy \, \left({E}_{a}\right)= m.g.l.\left(\text{cos}\beta -\text{cos}\alpha \right)\\ Charpy \, impact \, strenght= \frac{{E}_{a}}{{b}_{n}.h} ({\text{kJ}}/{\text{m}}^{2})\end{array}\right.$$

where $m$ is the hammer mass, $g$ is the gravitational acceleration, $l$ is the pendulum length, $\alpha$ is the angle of fall, $\beta$ is the angle of rise, ${E}_{a}$ is the fracture energy, ${b}_{n}$ is the width measured from the notch tip to the free edge and $h$ is the thickness of the specimen.

### Hardness test setup

EBP SH-D brand shore D hardness tester was used in the hardness test. Five specimens were produced for each test design. According to the standard, the specimen thickness must be at least 6 mm, and measurement locations should be spaced at least 6 mm apart. Hardness measurements were taken at five different points on each specimen, and the average of these measurements was recorded and reported. The Shore D hardness test employs an indenter with a sharp conical tip and a rigid spring. When pressure is applied to the material, the durometer measures the position of the indenter tip relative to the presser foot. The hardness value is then displayed after processing the data according to the correlation equation (Eq. 6) specified in the instrument manual.

6 $$Hardness \left(Shore D\right)=100-\left(L/0.025\right)$$

Here $L$ is the displacement of the indenter.

### Surface roughness test setup

Surface roughness measurements for the samples produced in each experimental design were performed using a Nanovea-ST400 3D optical surface profilometer. The part used in hardness measurement was selected as the surface roughness measurement part. The selected surface of the sample was scanned over an area of 1 mm^2^ using a 400 µm optical pen at a scanning speed of 1 µm/s. The average surface roughness value of the area on the scanned surface was obtained with the formula given in Eq. (7).

7 $${S}_{a}=\frac{1}{A}{\iint }_{A}\left|Z\left(x,y\right)\right|{d}_{x}{d}_{y}$$

where Sa is the average surface roughness value, A is the domain of definition and Z(x,y) is the ordinate values.

## Results and discussion

**Fig. 3:**
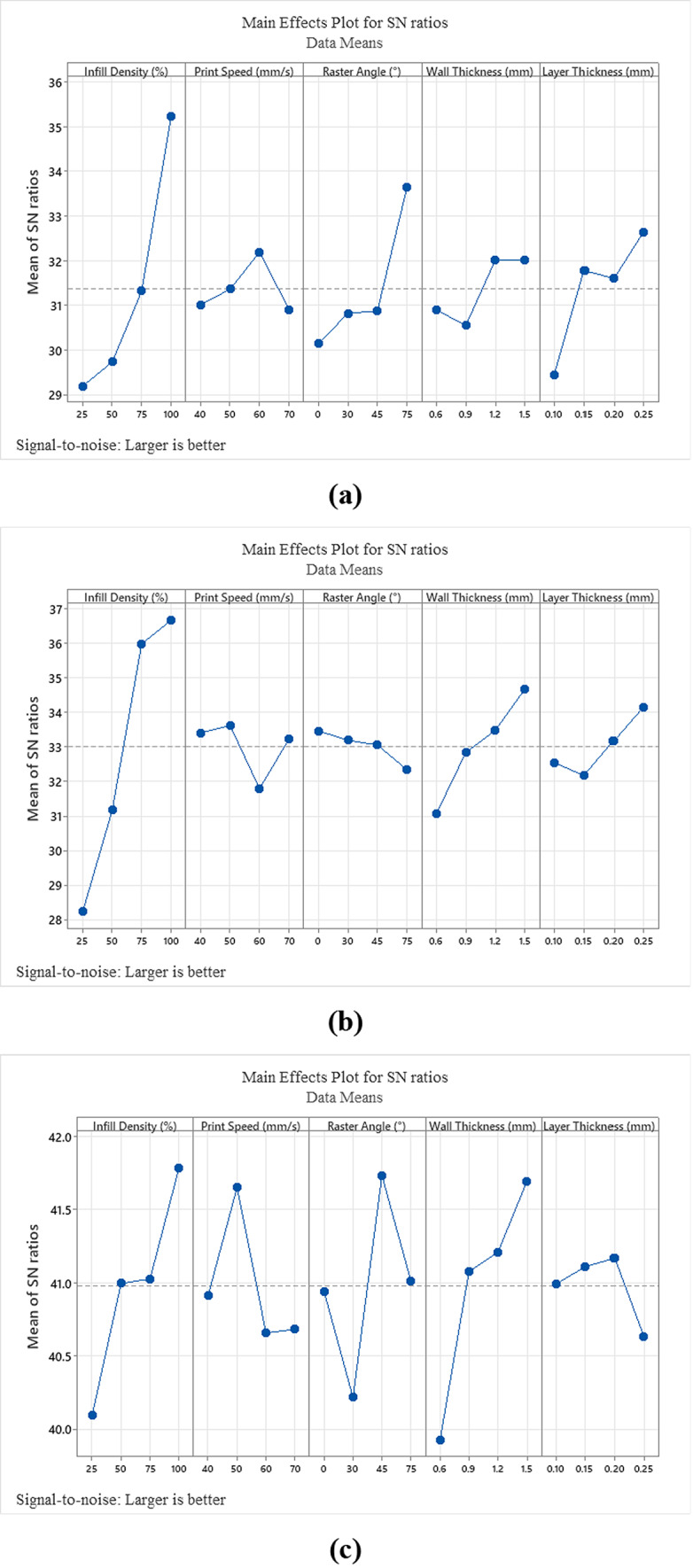

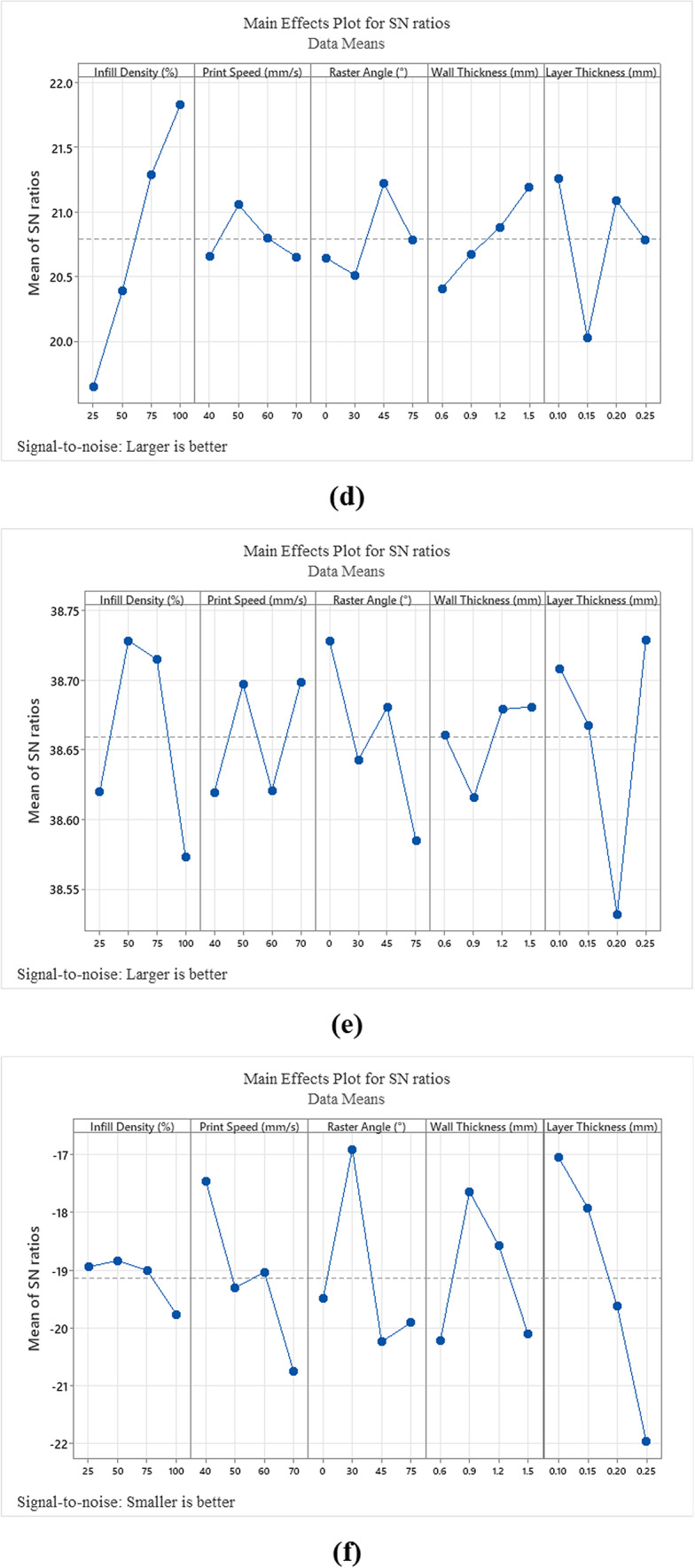
S/N rates for (**a**) tensile strength, (**b**) compression strength, (**c**) flexural strength, (**d**) Charpy impact strength, (**e**) hardness value and (**f**) surface roughness value.

In this study, the effects of thermoplastic PLA materials with different 3DP parameters on mechanical tests were investigated. The average values of the mechanical test results are listed in Table 3. Statistical analysis was performed using the Taguchi method. In general, the evaluation of mechanical test results by Taguchi method is done by interpreting S/N diagrams (Fig. 3). The S/N diagrams obtained in the research were realized and interpreted according to the "Larger is better" criterion. The "Larger is better" criterion finds the parameters that maximize the research output values.

**Table 3:** Average values of mechanical properties of tested specimens.

Test number	Tensile strength (MPa)	Compression strength (MPa)	Flexural strength (MPa)	Charpy impact strength (kJ/m^2^)	Hardness strength (Shore D)		Surface roughness (Sa)
1	18.59 27.2950	21.58	88.71 102.85	9.39	86.10		6.77 5.1167 9.8400 18.0333 7.2400 10.9733 9.3033 7.9133 8.1133 7.3167 10.7033 9.9567 7.8000 17.6667 6.5467 9.9600
2	27.29	25.14	102.85	8.67	85.18		5.11
3	33.00 46.2590	24.24	111.67	10.57	84.10		9.84
4	46.25 35.1260	33.81	102.47	9.89	85.87		18.03
5	35.12 29.1647	46.92	100.77	10.08	86.72		7.24
6	29.16 42.6440	50.33	134.14	11.50	86.40		10.97
7	42.64	21.12	97.64	9.17	85.37		9.30
8	20.21	34.65	119.90	11.26	87.05		7.91
9	35.79	72.64	134.49	11.52	86.37		8.11
10	42.33 36.0410	62.43	125.69	12.75	86.58		7.31
11	36.04 29.7203	64.36	105.15	11.26 10.96	86.82		10.70
12	29.72	53.61 65.86	90.31	10.96	85.23		9.95
13	66.75	65.86	126.62	12.41	82.12		7.80
14	61.73	66.86	123.42	12.80	86.13		17.66
15	52.74	69.23	118.09	13.23	85.02		6.54
16	55.81	70.31	123.42	11.07	86.20		9.96

### Tensile strength

The influences of factors and their interactions, as attained from ANOVA (fit general linear model) analysis, are detailed in Table 4. Furthermore, a fit regularization model analysis was performed to predict the research outcomes. Equation 8 presents the linear regression equation used to predict the tensile strength, with a 95% Confirmation Interval (CI). The algebraic effects of all 3DP process parameters on the tensile strength are also presented. The influence levels and S/N ratios for tensile strength are summarized in S1 Table 1. It was found that Infill Density and Raster Angle were the most significant parameters affecting tensile strength.

8 $$\begin{aligned} {\text{Tensile \,Stress }}\left( {{\text{MPa}}} \right) =\, & - {3}.{2 } + 0.{3529} \,{\text{Infill}}\, {\text{Density}} \,\left( \% \right) - 0.0{37}\, {\text{Printing}} \,{\text{Speed}}\, \left( {{\text{mm}}/{\text{s}}} \right) \\ & + 0.{1896} {\text{Raster}} \,{\text{Angle}} \,\left(^\circ \right) \, + {3}.{39}\, {\text{Wall}} \,{\text{Thickness}} \, \left( {{\text{mm}}} \right) + {67}.{4}\, {\text{Layer}} \,{\text{Thickness}} \left( {{\text{mm}}} \right) \\ \end{aligned}$$

**Table 4:** ANOVA of variance for S/N ratios on tensile test.

Source	DF	Seq SS	Contribution (%)	Adj SS	Adj MS	F-Value	*P*-value
Infill density (%)	1	1556.87	55.21	1556.87	1556.87	26.31	0.000
Printing speed (mm/s)	1	2.70	0.10	2.70	2.70	0.05	0.835
Raster angle (°)	1	420.63	14.92	420.63	420.63	7.11	0.024
Wall thickness (mm)	1	20.74	0.74	20.74	20.74	0.35	0.567
Layer thickness (mm)	1	227.08	8.05	227.08	227.08	3.84	0.079
Error	10	591.67	20.98	591.67	59.17		
Total	15	2819.69	100.00				

Looking at the contribution rates of the process parameters (Table 5), the Infill Density (55.21%), Raster Angle (14.92%) and Layer Thickness (8.05%) were found to be the most critical factors in determining the tensile strength values, respectively. The maximum Infill Density significantly affects the tensile stress^21,31,32^. It was observed that the stress value increased with increasing Infill Density^33^. Nonetheless, the surge in Infill Density increases the quantity of material used and printing times. Increases in the Raster Angle (0°—90°) have a negative effect on the tensile stress as they move the filament alignment away from the tensile axis^33^. The ANOVA test results (Table 4) and S/N ratios (Fig. 3) show that the MEX process parameters at level 4 of Infill Density, level 3 of Printing Speed, level 4 of Raster Angle, level 3 or 4 of Wall Thickness and level 4 of Layer Thickness were used to obtain maximum tensile strength values. When the best tensile strength prediction was produced with these process parameters, it was obtained that the S/N ratio was 40.2389 and the tensile strength was 75.6725 MPa.

**Table 5:** ANOVA for S/N ratios on compression test.

Source	DF	Seq SS	Contribution (%)	Adj SS	Adj MS	F-value	*P*-value
Infill density (%)	1	4543.00	80.86	4543.00	4543.00	121.42	0.000
Printing speed (mm/s)	1	58.31	1.04	58.31	58.31	1.56	0.240
Raster angle (°)	1	61.37	1.09	61.37	61.37	1.64	0.229
Wall thickness (mm)	1	509.65	9.07	509.65	509.65	13.62	0.004
Layer thickness (mm)	1	71.74	1.28	71.74	71.74	1.92	0.196
Error	10	374.17	6.66	374.17	37.42		
Total	15	5618.24	100.00				

**Fig. 4:**
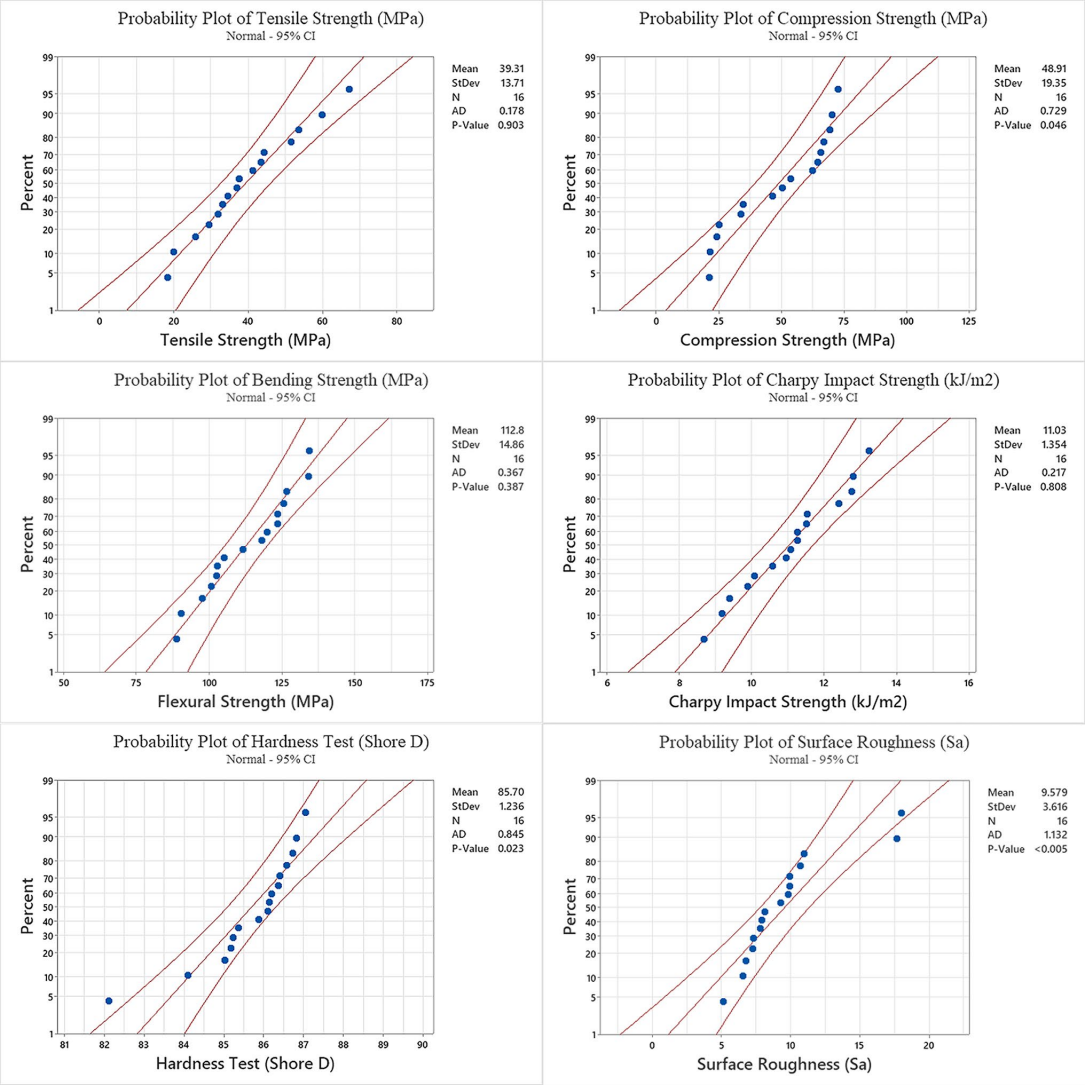
Probability plots of tensile, compression, flexural, Charpy impact, hardness and surface roughness tests.

The normal probability plot of tensile strength, derived from statistical analysis modeling, is shown in Fig. 4. The model accuracy is reflected in the distribution of data points on the graph.

The distribution of values close to the central axis in the probability graph indicates that the experimental study and the mathematical model are compatible.

### Compression strength

When the *p*-value of the ANOVA analysis (Table 5) for the compression test was taken into consideration, it was seen that the Infill Density and Wall Thickness parameters were statistically more significant. The result of linear regression analysis to predict the compression strength values is given in Eq. (9). The effect levels and S/N ratios for compression strength are shown in S2 Table 2. Infill Density and Wall Thickness were found to be the most effective parameters.

9 $$\begin{aligned} {\text{Compression\, Strength }}\left( {{\text{MPa}}} \right) =\, & - {1}.0 \, + \, 0.{6}0{\text{29 \,Infill\, Density }}\left( \% \right) - \, 0.{\text{171 \,Printing \,Speed }}\left( {{\text{mm}}/{\text{s}}} \right) \\ & - \, 0.0{\text{724\, Raster\, Angle }}\left(^\circ \right) \, + { 16}.{\text{83 \,Wall \,Thickness \,}}\left( {{\text{mm}}} \right) \, + { 37}.{\text{9 \,Layer \,Thickness }}\,\left( {{\text{mm}}} \right) \\ \end{aligned}$$

Figure 3 illustrates the levels of the items used in the S/N plot for compression strength. Additionally, it presents the effectiveness of the process parameters based on the "Larger is better" criterion, as analyzed by the Taguchi method. According to ANOVA results, it is seen that the contribution of Infill Density (80.86%) and Wall Thickness (9.07%) in compression strength is higher than the other parameters (Table 5). Tura et al., (2023) obtained a similar result in their study and found that Infill Density and Wall Thickness were the most effective parameters, respectively. Printing Speed, Raster Angle and Layer Thickness were also found to contribute around 1%. It is noticed that the impact of Infill Density on compressive strength as well as tensile strength is very important ^34^. Mangla et al., (2023) showed that both compressive and tensile strengths increased significantly with increasing Infill Density, while the surface roughness value decreased. Jaisingh Sheoran et al., (2020), in a similar study, observed that the internal structure tightens with increasing Infill Density and therefore the compression strength increases. Probability plots for compression strength according to Taguchi analysis results are given in Fig. 4. Upon reviewing the probability plots, it is clear that there is a good alignment between the experimental study and the mathematical model. The proximity of the graph distribution to the center axis indicates the model’s stability and accuracy. The balanced appearance of the distribution, as shown in Fig. 4, confirms the accuracy of the model. The ANOVA test results (Table 5) and S/N ratios (Fig. 3) show that the MEX process parameters at level 4 of Infill Density, level 2 of Printing Speed, level 1 of Raster Angle, level 4 of Wall Thickness and level 4 of Layer Thickness were used to obtain maximum compression strength values. When the best compression strength prediction was produced with these process parameters, it was obtained that the S/N ratio was 40.5055 and the tensile strength was 84.6175 MPa.

### Flexural strength

From the Taguchi analysis data outlined in Table 6, the factors with the highest contribution to flexural strength are Wall Thickness (28.40%) and Infill Density (25.66%). The effects of Printing Speed, Raster Angle and Layer Thickness range from approximately 0.9–3.6%. The linear regression equation used to predict flexural strength is provided in Eqs. (10). S3 Table 3 details the S/N ratios and impact levels for flexural strength. Wall Thickness and Infill Density were identified as the most influential parameters affecting flexural strength.

10 $$\begin{aligned} {\text{Flexural \,Strength }}\,\left( {{\text{MPa}}} \right) =\, & {88}.{9 } + \, 0.{\text{261 \,Infill \,Density }}\left( \% \right) - 0.{\text{242 \,Printing \,Speed }} \, \left( {{\text{mm}}/{\text{s}}} \right) \\ & + 0.0{\text{53 \,Raster \,Angle }}\left(^\circ \right) + { 22}.{\text{86 \,Wall \,Thickness }}\left( {{\text{mm}}} \right) \, - { 28}.{\text{7 \,Layer \,Thickness }}\left( {{\text{mm}}} \right) \\ \end{aligned}$$

**Table 6:** ANOVA for S/N ratios on flexural strength.

Source	DF	Seq SS	Contribution (%)	Adj SS	Adj MS	F-value	*P*-value
Infill density (%)	1	850.08	25.66	850.08	850.08	6.39	0.030
Printing speed (mm/s)	1	117.57	3.55	117.57	117.57	0.88	0.369
Raster angle (°)	1	32.67	0.99	32.67	32.67	0.25	0.631
Wall thickness (mm)	1	940.91	28.40	940.91	940.91	7.07	0.024
Layer thickness (mm)	1	41.16	1.24	41.16	41.16	0.31	0.590
Error	10	1330.79	40.17	1330.79	133.08		
Total	15	3313.18	100.00				

According to Table 6, Infill Density and Wall Thickness were identified as statistically significant parameters. The parameter with the least contribution is Raster Angle (0.99%) and the parameters with the highest contribution are Wall Thickness (28.40%) and Infill Density (25.66%). The levels of the factors used in the S/N plot for compression strength are shown in Fig. 3. Burge et al., (2020) found the order of contribution of the parameters in a similar way. In the study, it was noticed that continuous increase in Infill Density and Wall Thickness had a positive effect on flexural strength (Fig. 3). Considering the flexural strength, the contribution of Printing Speed was found to be higher than the Layer Thickness (Table 6) ^35^. It is thought that the Layer Thickness is inversely proportional to the printing time, and it would be appropriate to balance this with the Printing Speed. Kechagias (2024) also found that the effect of infill density on flexural strength was significant. Probability plots for flexural strength are given in Fig. 4. Considering the probability plot, it is seen that the mathematical model and the experimental study are compatible. Considering the ANOVA test results (Table 6) and S/N ratios (Fig. 3), the maximum flexural strength value is obtained when the Infill Density is at level 4, Printing Speed at level 2, Raster Angle at level 3, Wall Thickness at level 4 and Layer Thickness at level 3. In addition, when the flexural strength prediction was produced with these parametric values, it was obtained that the S/N ratio was 44.1325 and the flexural strength was 153.430 MPa. The maximum flexural strength values were higher than those in similar studies^36,37^.

### Charpy impact strength

**Fig. 5:**
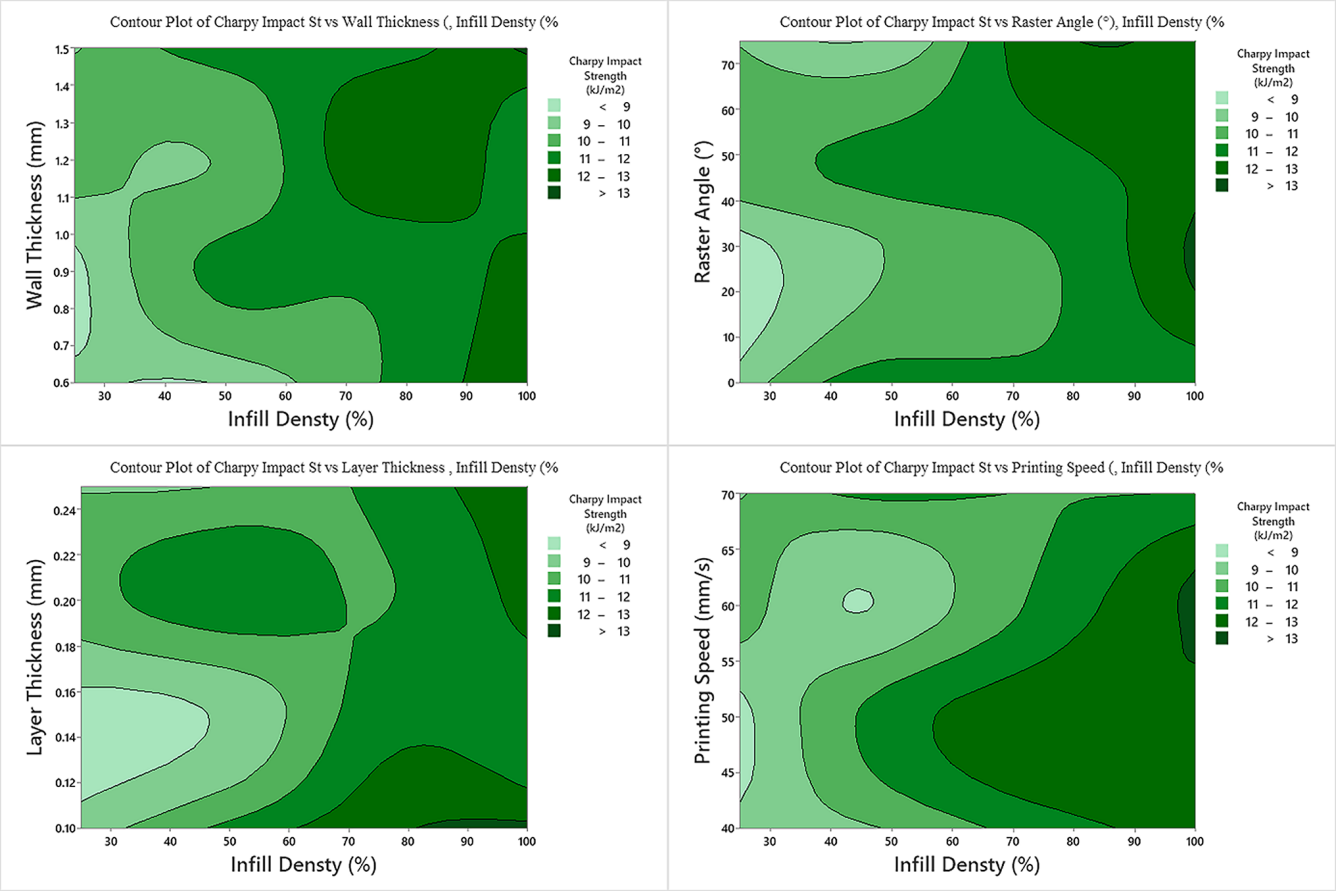
Impact of infill density and other parameters on Charpy impact strength.

According to the Charpy Impact strength, the parameter with the highest contribution is the Infill Density (63.61%) (Table 7). The others were Wall Thickness (6.92%), Raster Angle (1.16%), Layer Thickness (0.37%) and Printing Speed (0.21%), respectively. stated that Infill Density is an important factor in the impact strength of PLA materials and that the impact strength will increase with increasing Infill Density. In our research, it was found that the contribution of the Infill Density to the impact strength was the highest and its interaction with other parameters on the impact strength is presented in Fig. 5. With the increase in Infill Density, the contact of the molten filaments with each other increased and the interconnectivity became better. This increases impact strength^38^. The result of linear regression analysis to predict the Charpy impact strength is given in Eq. (11). S4 Table 4 shows the S/N ratios and impact levels for Charpy Impact Strength. Infill Density was found to be the first and Layer Thickness was found to be the second effective parameter. Atakok et al. (2022) reported that Layer Thickness and Infill Density have marked effects on impact strength.

11 $$\begin{aligned} {\text{Charpy\, Impact\, Strength }}\left( {{\text{kJ}}/{\text{m}}^{{2}} } \right) \, = \,& { 7}.{97 } + \, 0.0{\text{3741 Infill\, Density }}\left( \% \right) - 0.00{\text{54 \,Printing\, Speed }}\left( {{\text{mm}}/{\text{s}}} \right) \\ & + \, 0.00{\text{521 Raster\, Angle }}\left(^\circ \right) \, + { 1}.0{\text{28\, Wall\, Thickness }}\left( {{\text{mm}}} \right) \, - { 1}.{\text{43 \,Layer\, Thickness }}\left( {{\text{mm}}} \right) \\ \end{aligned}$$

**Table 7:** ANOVA of S/N ratios for Charpy impact test.

Source	DF	Seq SS	Contribution (%)	Adj SS	Adj MS	F-value	*P*-value
Infill density (%)	1	17.4971	63.61	17.4971	17.4971	22.93	0.001
Printing speed (mm/s)	1	0.0585	0.21	0.0585	0.0585	0.08	0.788
Raster angle (°)	1	0.3179	1.16	0.3179	0.3179	0.42	0.533
Wall thickness (mm)	1	1.9026	6.92	1.9026	1.9026	2.49	0.145
Layer thickness (mm)	1	0.1017	0.37	0.1017	0.1017	0.13	0.723
Error	10	7.6312	27.74	7.6312	0.7631		
Total	15	27.5090	100.00

The parameters with the least contribution were observed as Layer Thickness Printing Speed, and Raster Angle. The parameter levels providing the maximum Charpy impact strength were obtained as Infill Density level 4, Printing Speed level 2, Raster Angle level 3, Wall Thickness level 4 and Layer Thickness level 1 (Fig. 3). When the prediction was generated according to these determined values, the S/N value was found to be 23.3990 and the Charpy impact stress value was found to be 14.4063 kJ/m^2^. Probability plot for Charpy impact strength is given in Fig. 4. The probability plot demonstrates that the experimental study aligns with the mathematical model.

When the graph is analyzed, the interactions of the Infill Density and other parameters on the impact strength appear to be similar. Impact strength increases with increasing Infill Density and other parameters. When designing and manufacturing, not only the contribution of parameters is important. Parameters such as printing time, amount of filament consumed, etc. should also be examined. For example, as the Infill Density increases, the printing time and the amount of filament increases, which affects both the time spent and the cost.

### Hardness measurement

Table 8 presents the results of ANOVA for S/N ratios in the hardness measurement test. It was observed that the parameter with the highest contribution rate in hardness measurement was the Raster Angle (14.67%). The parameters with low contribution rates were found to be Printing Speed (2.06%), Infill Density (1.89%), Wall Thickness (1.30%) and Layer Thickness (0.46%). The equation obtained from linear regression analysis to predict the hardness measurement is given in Eq. (12). S5 displays the S/N ratios and effect levels for hardness measurement. The parameters were ranked by their impact on hardness as follows: Layer Thickness, Infill Density, Raster Angle, Printing Speed, and Wall Thickness, from most to least significant. Mani et al. (2022) also identified Layer Thickness and Infill Density as the most influential parameters in their study.

12 $$\begin{aligned} {\text{Hardness\, measurement }}\left( {\text{Shore D}} \right) \, = \,& {85}.{69 } - \, 0.00{\text{59 Infill \,Density }}\left( \% \right) \, + \, 0.0{\text{154\, Printing\, Speed }}\left( {{\text{mm}}/{\text{s}}} \right) - 0.0{\text{169 Raster \,Angle }}\left(^\circ \right) \\ & + \, 0.{\text{41\, Wall \,Thickness }}\left( {{\text{mm}}} \right) \, - { 1}.{\text{45 \,Layer\, Thickness }}\left( {{\text{mm}}} \right) \\ \end{aligned}$$

**Table 8:** ANOVA of S/N ratios for Hardness measurement.

Source	DF	Seq SS	Contribution (%)	Adj SS	Adj MS	F-value	*P*-value
Infill density (%)	1	0.4337	1.89	0.4337	0.4337	0.24	0.636
Printing speed (mm/s)	1	0.4718	2.06	0.4718	0.4718	0.26	0.622
Raster angle (°)	1	3.3601	14.67	3.3601	3.3601	1.84	0.205
Wall thickness (mm)	1	0.2981	1.30	0.2981	0.2981	0.16	0.695
Layer thickness (mm)	1	0.1049	0.46	0.1049	0.1049	0.06	0.815
Error	10	18.2387	79.62	18.2387	1.8239		
Total	15	22.9071	100.00				

According to the S/N diagram, the parameters and levels giving the highest hardness measurement value were found to be Infill Density level 2, Printing Speed level 4, Raster Angle level 1, Wall Thickness level 4 and Layer Thickness level 4 (Fig. 3). When the hardness measurement was estimated according to these values, it was seen that the S/N was 38.9284 and the hardness measurement was 88.335 shore D. Probability plot for hardness measurement is given in Fig. 4. The probability plot indicates that the experimental study aligns well with the mathematical model. Additionally, it was noted that while some values fell outside the graph, they did not significantly impact the overall system.

### Surface roughness measurement

S/N graph of surface roughness measurement is given in Table 9. It was observed that the parameters with high contribution were Layer Thickness (42.17%) and Printing Speed (11.85%), while the parameters with low contribution were Raster Angle (2.98%), Infill Density (0.34%) and Wall Thickness (0.05%). Equation (13) shows the linear regression analysis equation for surface roughness measurement. The level of influence of the parameters on surface roughness is shown in S6 Table 6.

13 $$\begin{aligned} {\text{Surface Roughness }}\left( {{\text{Sa}}} \right) \, =\, & - {5}.00 \, + \, 0.00{\text{73 Infill Density }}\left( \% \right) \, + \, 0.{1}0{\text{78 Printing Speed }}\left( {{\text{mm}}/{\text{s}}} \right) \, + \, 0.0{\text{224 Raster Angle }}\left(^\circ \right) \, \\ & + \, 0.{\text{23 Wall Thickness }}\left( {{\text{mm}}} \right) \, + { 4}0.{\text{7 Layer Thickness }}\left( {{\text{mm}}} \right) \\ \end{aligned}$$

**Table 9:** ANOVA S/N ratios for Surface roughness measurement.

Source	DF	Seq SS	Contribution (%)	Adj SS	Adj MS	F-value	*P*-value
Infill density (%)	1	0.664	0.34	0.6643	0.6643	0.08	0.784
Printing speed (mm/s)	1	23.252	11.85	23.2525	23.2525	2.78	0.126
Raster angle (°)	1	5.852	2.98	5.8525	5.8525	0.70	0.422
Wall thickness (mm)	1	0.093	0.05	0.0927	0.0927	0.01	0.918
Layer thickness (mm)	1	82.736	42.17	82.7363	82.7363	9.90	0.010
Error	10	83.584	42.61	83.5838	8.3584		
Total	15	196.182	100.00				

According to Fig. 3, the parameters and levels that achieve the maximum surface roughness are as follows: Infill Density at level 2, Printing Speed at level 1, Raster Angle at level 2, Wall Thickness at level 2, and Layer Thickness at level 1. With these settings, the predicted maximum surface roughness and S/N values are 0.2608 Sa and − 11.3481, respectively. S6 Table 6 shows that the influence on surface roughness is ranked as Layer Thickness, Raster Angle, Printing Speed, Wall Thickness, and Infill Density. Mani et al., (2022) identified Layer Thickness as the most significant parameter affecting surface roughness. In a similar study, significant changes in the surface roughness value Ra were observed with sudden changes in layer thickness^39^. Figure 3 demonstrates that surface roughness increases with Layer Thickness, attributed to the larger gaps formed between filaments as filament thickness increases.

## Conclusions

This research concentrated on optimizing 3DP parameters to enhance the tensile, compression, flexural, hardness, impact strengths, and surface roughness values of PLA materials. Through optimization studies and comprehensive statistical and parametric analyses, the following results were obtained:

- The effect of the Infill Density parameter on tensile, compressive and impact strength was the best and the highest contribution rate (55.21%, 80.86% and 63.61%). Begum et al. (2024) emphasize the potential of high infill density (particularly 60%) to enhance the overall strength of printed parts and report that this condition increases the energy absorption capacity of the component. In flexural strength, the order of effect and contribution rate (25.66%) were found in the 2nd place. High connections between filaments had a positive effect on the strengths. The effect of Infill Density on hardness test was ranked 2nd and its contribution was found to be low (1.89%). Şirin et al. (2023) highlight in their presented data on the effect of infill density in hardness testing that the hardness measurement probes do not penetrate sufficiently into the part, resulting in a limited contribution to hardness. The effect (5) and contribution (0.34%) on surface roughness were low. Since the Infill Density represents the geometric density within the part, its positive effect on the part surface was found to be low. In terms of surface roughness, the effect of infill density is generally low. Studies have observed that increasing infill density does not lead to a significant improvement in surface roughness^40^. In all tests except the hardness test, it was observed that the effect values increased as the Infill Density levels increased.
- The effect ranking (5, 4, 4, 5 and 4) and contribution ratios (%0.1, %1.04, %3.55, 0.21 and %2.06) of the Printing Speed parameter on tensile, compression, flexural, impact strength and hardness were found to be lower than the other parameters. Pandžić et al. (2023) found that while printing speed does influence tensile properties of FDM-printed carbon fiber-reinforced polyamide composites, its effect is less pronounced compared to other parameters like layer height and infill density. It was observed that only the effect ranking on surface roughness was 3 and the contribution percentage was 11.85%. It was found that the only test in which the effect and contribution of the Printing Speed parameter was significant was surface roughness. In the study conducted by Golhin et al. (2023), they emphasize that printing speed is a critical parameter affecting the surface quality of polymeric parts, noting that slower printing speeds generally result in lower surface roughness.
- The effect of the Raster Angle parameter was high in tensile (2) and surface roughness (2) tests, and its contribution was high in tensile (14.92%) and hardness (14.67%) tests. Due to the high number of parameters (5) in the tests, the effect and contribution rates were observed to be low. If the number of parameters were lower, the values of the effect and contribution rates would be different. When the S/N ratios of the Raster Angle in all tests (Fig. 3) are examined, it is observed that there is no linear situation.
- The effectiveness of the Wall Thickness parameter in compression and flexural tests (2 and 1) and its contribution to compression, flexural and impact tests (9.07%, 28.4% and 6.97%) were found to be high. A study by Passari et al. (2024) emphasized that parameters closely related to wall thickness, such as layer thickness and wall perimeter, significantly influence the compression resistance of 3D-printed parts. Since the Wall Thickness parameter represents the portion of the part close to the surface at 100% fullness, its importance in compression, flexural and impact tests was found to be high.
- Layer Thickness appears to be the second most important parameter after Infill Density. The effect on impact, hardness and surface roughness tests (2, 1 and 1) is significant. Increased layer thickness leads to poorer adhesion between layers, resulting in diminished impact strength. Taranath et al. (2024) observed in their study that impact resistance values decreased from 9.5 to 5.5 J/mm^2^ with thicker layers. It was observed that the distance value between the filaments increased with increasing Layer Thickness and thus the surface roughness value was high. The effect values in tensile and compression were found to be moderate (3).

As a result, it was seen that the effect and contribution rates of Infill Density and Layer Thickness parameters on mechanical tests in 3DP processes were significant. In future research, separate and detailed evaluations of the parameters and tests that are considered important in this study can be carried out separately and in detail to provide healthier and more meaningful results.

### Potential areas for future research

In the present research, the thermoplastic PLA material generally used in the MEX method was chosen. There are different homogeneous and composite materials used in this production method and their evaluation will be interesting for the future of the research. In future research, the interactions of other parameters used in the 3DP process (Nozzle diameter, Raster width, Air gap, Infill pattern, etc.) on mechanical strengths can be investigated. The scalability of these materials to larger part sizes, production and feasibility studies can be investigated.

### Limitations of the study

In the research, only five factors and four levels in each factor were used as 3DP processes. These factors may not be sufficient to examine the MEX method and the design strengths generated by this method. Only most of the mechanical tests were used in the research. In addition to these, the research can be extended with other mechanical tests (Abrasion test, Izod test, etc.) and thermal analysis. In addition, the fracture surfaces of the parts in the test results can be interpreted by examining them under a microscope and the research can be detailed.

## Electronic supplementary material

Below is the link to the electronic supplementary material.

Supplementary Material 1

Supplementary Material 2

Supplementary Material 3

Supplementary Material 4

Supplementary Material 5

Supplementary Material 6

## Data Availability

The datasets used and/or analysed during the current study available from the corresponding author on reasonable request.
