# Supplementary material for: Investigation of the effects of 3D printing parameters on mechanical tests of PLA parts produced by MEX 3D printing using Taguchi method
Source: Sci Rep. 2025 Apr 29;15:15008. doi: 10.1038/s41598-025-98832-0 (PMC12041565; doi:10.1038/s41598-025-98832-0)
Supplement: Supplementary file 1 — Supplementary Material 1 [file 41598_2025_98832_MOESM1_ESM.docx]

| **Table S1.** Signal to Noise Ratios (Larger is better) for Tensile Strength. | | | | | |
| --- | --- | --- | --- | --- | --- |
| **Level** | **Infill Density (%)** | **Print Speed (mm/s)** | **Raster Angle (°)** | **Wall Thickness (mm)** | **Layer Thickness (mm)** |
| 1 | 29.19 | 31.02 | 30.14 | 30.90 | 29.45 |
| 2 | 29.73 | 31.36 | 30.82 | 30.55 | 31.78 |
| 3 | 31.32 | 32.19 | 30.87 | 32.01 | 31.60 |
| 4 | 35.23 | 30.91 | 33.63 | 32.01 | 32.65 |
| Delta | 6.04 | 1.28 | 3.49 | 1.46 | 3.19 |
| Rank | 1 | 5 | 2 | 4 | 3 |
