# Supplementary material for: Investigation of the effects of 3D printing parameters on mechanical tests of PLA parts produced by MEX 3D printing using Taguchi method
Source: Sci Rep. 2025 Apr 29;15:15008. doi: 10.1038/s41598-025-98832-0 (PMC12041565; doi:10.1038/s41598-025-98832-0)
Supplement: Supplementary file 2 — Supplementary Material 2 [file 41598_2025_98832_MOESM2_ESM.docx]

| **Table S2.** Signal to Noise Ratios (Larger is better) for Compression Strength. | | | | | |
| --- | --- | --- | --- | --- | --- |
| **Level** | **Infill Density (%)** | **Print Speed (mm/s)** | **Raster Angle (°)** | **Wall Thickness (mm)** | **Layer Thickness (mm)** |
| 1 | 28.24 | 33.40 | 33.46 | 31.06 | 32.55 |
| 2 | 31.17 | 33.61 | 33.18 | 32.83 | 32.16 |
| 3 | 35.97 | 31.79 | 33.05 | 33.47 | 33.17 |
| 4 | 36.65 | 33.22 | 32.34 | 34.66 | 34.15 |
| Delta | 8.42 | 1.83 | 1.12 | 3.60 | 1.99 |
| Rank | 1 | 4 | 5 | 2 | 3 |
