# Supplementary material for: Investigation of the effects of 3D printing parameters on mechanical tests of PLA parts produced by MEX 3D printing using Taguchi method
Source: Sci Rep. 2025 Apr 29;15:15008. doi: 10.1038/s41598-025-98832-0 (PMC12041565; doi:10.1038/s41598-025-98832-0)
Supplement: Supplementary file 3 — Supplementary Material 3 [file 41598_2025_98832_MOESM3_ESM.docx]

| **Table S3. Signal to Noise Ratios (Larger is better) for Flexural Strength.** | | | | | |
| --- | --- | --- | --- | --- | --- |
| **Level** | **Infill Density (%)** | **Print Speed (mm/s)** | **Raster Angle**  **(°)** | **Wall Thickness (mm)** | **Layer Thickness (mm)** |
| 1 | 40.09 | 40.91 | 40.94 | 39.92 | 40.99 |
| 2 | 41.00 | 41.65 | 40.22 | 41.08 | 41.11 |
| 3 | 41.03 | 40.66 | 41.73 | 41.21 | 41.17 |
| 4 | 41.79 | 40.68 | 41.01 | 41.70 | 40.64 |
| Delta | 1.69 | 0.99 | 1.52 | 1.77 | 0.53 |
| Rank | 2 | 4 | 3 | 1 | 5 |
