# Supplementary material for: Investigation of the effects of 3D printing parameters on mechanical tests of PLA parts produced by MEX 3D printing using Taguchi method
Source: Sci Rep. 2025 Apr 29;15:15008. doi: 10.1038/s41598-025-98832-0 (PMC12041565; doi:10.1038/s41598-025-98832-0)
Supplement: Supplementary file 4 — Supplementary Material 4 [file 41598_2025_98832_MOESM4_ESM.docx]

| **Table S4.** Signal to Noise Ratios (Larger is better) for Charpy Impact Strength. | | | | | |
| --- | --- | --- | --- | --- | --- |
| **Level** | **Infill Density (%)** | **Print Speed (mm/s)** | **Raster Angle**  **(°)** | **Wall Thickness (mm)** | **Layer Thickness (mm)** |
| 1 | 19.65 | 20.66 | 20.65 | 20.41 | 21.26 |
| 2 | 20.39 | 21.06 | 20.51 | 20.68 | 20.03 |
| 3 | 21.29 | 20.80 | 21.22 | 20.88 | 21.09 |
| 4 | 21.83 | 20.65 | 20.78 | 21.20 | 20.78 |
| Delta | 2.18 | 0.41 | 0.71 | 0.78 | 1.23 |
| Rank | 1 | 5 | 4 | 3 | 2 |
