# Supplementary material for: Investigation of the effects of 3D printing parameters on mechanical tests of PLA parts produced by MEX 3D printing using Taguchi method
Source: Sci Rep. 2025 Apr 29;15:15008. doi: 10.1038/s41598-025-98832-0 (PMC12041565; doi:10.1038/s41598-025-98832-0)
Supplement: Supplementary file 5 — Supplementary Material 5 [file 41598_2025_98832_MOESM5_ESM.docx]

| **Table S5.** Signal to Noise Ratios (Larger is better) for Hardness Measurement. | | | | | |
| --- | --- | --- | --- | --- | --- |
| **Level** | **Infill Density (%)** | **Printing Speed (mm/s)** | **Raster Angle**  **(°)** | **Wall Thickness (mm)** | **Layer Thickness (mm)** |
| 1 | 38.62 | 38.62 | 38.73 | 38.66 | 38.71 |
| 2 | 38.73 | 38.70 | 38.64 | 38.62 | 38.67 |
| 3 | 38.71 | 38.62 | 38.68 | 38.68 | 38.53 |
| 4 | 38.57 | 38.70 | 38.58 | 38.68 | 38.73 |
| Delta | 0.16 | 0.08 | 0.14 | 0.07 | 0.20 |
| Rank | 2 | 4 | 3 | 5 | 1 |
