# Supplementary material for: Investigation of the effects of 3D printing parameters on mechanical tests of PLA parts produced by MEX 3D printing using Taguchi method
Source: Sci Rep. 2025 Apr 29;15:15008. doi: 10.1038/s41598-025-98832-0 (PMC12041565; doi:10.1038/s41598-025-98832-0)
Supplement: Supplementary file 6 — Supplementary Material 6 [file 41598_2025_98832_MOESM6_ESM.docx]

| Table 6. Signal to Noise Ratios (Smaller is better) for Surface Roughness Measurement | | | | | |
| --- | --- | --- | --- | --- | --- |
| **Level** | **Infill Density (%)** | **Print Speed (mm/s)** | **Raster Angle (°)** | **Wall Thickness (mm)** | **Layer Thickness (mm)** |
| 1 | -18.94 | -17.46 | -19.49 | -20.22 | -17.05 |
| 2 | -18.84 | -19.30 | -16.91 | -17.64 | -17.93 |
| 3 | -19.01 | -19.04 | -20.24 | -18.58 | -19.62 |
| 4 | -19.77 | -20.75 | -19.91 | -20.11 | -21.96 |
| Delta | 0.93 | 3.29 | 3.32 | 2.58 | 4.91 |
| Rank | 5 | 3 | 2 | 4 | 1 |
